# Foreign‐Trained Dentists' Experiences of the Swedish Proficiency Test for Recognition of Dental Qualifications

**DOI:** 10.1111/eje.70124

**Published:** 2026-02-27

**Authors:** Jesper Dalum, Joséphine Brodén, Annsofi Johannsen, Gunilla Sandborgh‐Englund

**Affiliations:** ^1^ Department of Dental Medicine Karolinska Institutet Stockholm Sweden

## Abstract

**Background:**

Dentists educated outside the EU/EEA can undergo a national proficiency test to obtain a Swedish dental licence. These assessments aim to ensure competence and patient safety, but little is known about how foreign‐trained dentists experience the process. This study aimed to describe the experiences of dentists undertaking the theoretical and clinical skills examinations included in the Swedish proficiency test for dentists educated outside EU/EEA.

**Methods:**

Written responses to open‐ended questions from post‐examination online surveys were analysed, using systematic text condensation. Surveys were collected after six theoretical examinations (*n* = 486) and four clinical skills examinations (*n* = 190) between 2022 and 2024. Respondents represented 54 countries.

**Results:**

Participants' experiences were summarised in one core theme: challenges in demonstrating competence, divided into four subthemes: (a) preparing for the exams, (b) relevance of assessments, (c) language barriers and (d) stress.

**Discussion:**

Participants described several challenges in demonstrating competence during the recognition process, mirroring experiences previously reported by dentists and other health‐care professionals in similar high‐stakes contexts. Participants also expressed a need for preparatory guidance, language support and tasks closely aligned with professional practice, which may be useful in the future development of recognition processes for dentists and health professionals seeking to work under a licence within the European Union.

**Conclusion:**

Participants confirmed known challenges in the recognition process while also highlighting a novel concern: they described the theoretical and clinical examinations, modelled on undergraduate assessments, as designed for students and as non‐representative of real clinical situations, which they described as hindering their ability to demonstrate competence in professional practice.

## 1 Introduction

The recognition of dental qualifications obtained outside the European Union varies across member states, but the overall goal is consistent: to ensure that all dentists working within the European Union meet the required standards of competence and thereby safeguard patient safety [1]. Since 2017, the Swedish National Board of Health and Welfare has implemented proficiency tests for the recognition of healthcare professionals educated outside the EU/EEA [2]. The proficiency tests are crucial for verifying that participating dentists possess the necessary skills and knowledge to practice safely and effectively under a licence. Each test comprises a theoretical and a clinical skills examination, followed by a 6‐month clinical practice period [2]. The Swedish proficiency test is not designed as an educational pathway, but as a high‐stakes assessment to determine whether candidates meet the requirements for progression within the licensure process. The present study focuses on dentists who have participated in the theoretical and clinical skills examinations included in the proficiency test for dentists educated outside EU/EEA.

Each year, over three hundred participants take part in the theoretical examination, and almost two hundred take part in the clinical skills examination; both are held at Karolinska Institutet [3]. The theoretical examination is a computer‐based examination, primarily featuring case‐based single‐best‐answer questions designed to assess both knowledge and application of knowledge. After passing the theoretical examination, participants can proceed to the clinical skills examination, which consists of operative tasks on a dental manikin and Observed Structured Clinical Examination type stations (OSCE), aiming to assess clinical and communication skills. The clinical skills examinations are conducted over 2 days. Both the theoretical and clinical skills examination are aligned with the learning outcomes of the degree in dental surgery [4] and current undergraduate curricula, assuring that a relevant and representative sample of knowledge and skills is assessed before progression to the 6‐month clinical practice period.

Although proficiency tests have been conducted in Sweden for almost a decade in its current form, much remains unknown about third country health‐care professionals' experiences of the proficiency examinations within the recognition process. Two previous studies of foreign trained dentists in Sweden have shown that an increase in age was associated with lower scores in theoretical examination scores and a higher risk of failing the clinical skills examination, especially in tasks involving communication [5, 6]. These findings suggest that clinical experience may not be as indicative of passing the examinations as expected. Australian studies on dentists' experiences of recognition examinations have identified several individual and structural barriers, such as language and communication difficulties, financial and time‐related costs, high perceived stress associated with examinations, lack of preparatory support and challenges related to settlement and professional integration [7, 8, 9, 10]. However, these studies were conducted almost a decade ago and outside the European Union. Only a handful of studies concerning recognition processes have been conducted in Sweden, but none involve dentists [11, 12, 13, 14, 15, 16, 17]. The more recent studies of recognition related issues from Sweden highlight barriers, such as language difficulties, origin of education and the need for better preparatory support [16, 17]. These studies were, however, conducted in other contexts that varied in terms of profession and system of assessment.

Recently, the European Commission has identified that the recognition of skills and qualifications for third‐country nationals is a slow and complex process in many member states [18]. The Commission therefore recommends all member states to reduce barriers in recognition, adopting a ‘skills first’ approach, combining the recognition of qualifications with an assessment of skills and experiences wherever possible [18]. It is of importance to analyse the experiences of foreign trained dentists during the recognition process in order to identify challenging or unclear parts of the process and allow for the improvement of assessment efficiency. Their experiences may also contribute to relevance, fairness and to improve the validity of the process [19]. Given the varying recertification processes across countries, professionals trained outside the European Union often face significant challenges. Although several studies have examined the challenges faced by foreign‐trained healthcare professionals in diverse contexts, there is a noticeable gap in the literature regarding the specific experiences of foreign‐trained dentists in Europe and in Sweden. This study aimed to describe the experiences of third‐country dentists undertaking the theoretical and clinical skills examinations as part of the Swedish recognition process for dentists educated outside EU/EES.

## 2 Materials and Methods

Data were collected through an online survey administered after participants completed either a theoretical or a clinical skills examination. The survey was initially developed in 2017 to gather participant feedback and suggestions for exam improvements. In this study, we focused on the written responses to open‐ended questions, providing participants' experiences of the proficiency test. A qualitative approach was employed, utilising systematic text condensation for analysis, as outlined by Malterud [20]. The feedback was analysed to identify recurring themes and gain insights into the dentists' experiences and perceptions of both the theoretical and clinical components of the assessments.

### 2.1 Study Context

The study was conducted at Karolinska Institutet. At the time of the study, the theoretical examination was held three times per year, and the clinical skills examination two times per year. The licensure process is sequential: candidates must first pass the theoretical examination before being eligible to proceed to the clinical skills examination. Following successful completion of the theoretical and clinical skills examinations, candidates undertake a 6‐month period of supervised clinical practice at a dental clinic. During this period, professional practice is assessed through workplace‐based assessments linked to the national intended learning outcomes for the degree of dental surgery. Although the proficiency test is administered by a university, it is conducted at the request of the National Board of Health and Welfare [2]. Within this mandate, the proficiency test does not include the provision of training or education but serves as an assessment within the licensure process, forming part of the documentation used by the National Board of Health and Welfare when deciding on licensure. The Swedish proficiency test does not require candidates to meet formal Swedish language proficiency requirements prior to participation. Language proficiency is instead assessed later in the licensure process and constitutes a requirement for licensure by the National Board of Health and Welfare. As part of the proficiency test procedure, candidates also complete a mandatory course in Swedish laws and regulations. Approximately 100 individuals participated in each theoretical examination and 75 in each clinical skills examination. After each examination, all participants received an online survey with questions about their experiences of the exam.

### 2.2 Participants

Respondents were recruited consecutively after each examination during the period 2022–2024. The post‐examination survey was anonymous, and background variables such as age and sex were not collected. Previously published data from the Swedish proficiency test showed that in theoretical examinations conducted 2018–2019, 47% of participants were female (*n* = 316), with a median age of 34 years (IQR 30–42). Most participants (79%) had completed dental programmes of at least five years' duration [5]. Corresponding data from the clinical skills examinations conducted 2018 to 2022 show that 56% of participants were female (*n* = 181), with a median age of 34 years (IQR 30–40) [6].

Participation in the post‐examination survey was voluntary. At the end of the survey, participants were given the option to consent to the use of their answers in research. Only respondents who gave consent were included. A total of 486 individual surveys were collected from six theoretical examinations between 2022 and 2024. The overall response rate for the six surveys was 74% (Highest 79%, Lowest 70%). From the clinical skills examinations, 190 surveys from four examinations were collected during the same period. The overall response rate for the clinical skills examination was 59.5% (Highest 69%, Lowest 39%). Self‐reported median years in the profession was 5 years (0–36 years), and 54 countries were represented among the respondents.

### 2.3 The Survey

The survey was originally created to gather the participants' experiences and suggestions for improvement. Both Likert scale questions (rating agreement from strongly agree to strongly disagree) and open‐ended questions were included to allow participants to freely express their thoughts and experiences of the exams. The surveys were presented in both Swedish and English (See Data S1). There were four open‐ended questions in the survey after the theoretical examination and six after the clinical skills examination. An overview of the open‐ended questions and the number of respondents per question is presented in Figure 1. The open‐ended questions did not have any word limit. Only answers from the open‐ended questions were collected and analysed in the present study.

**FIGURE 1:**
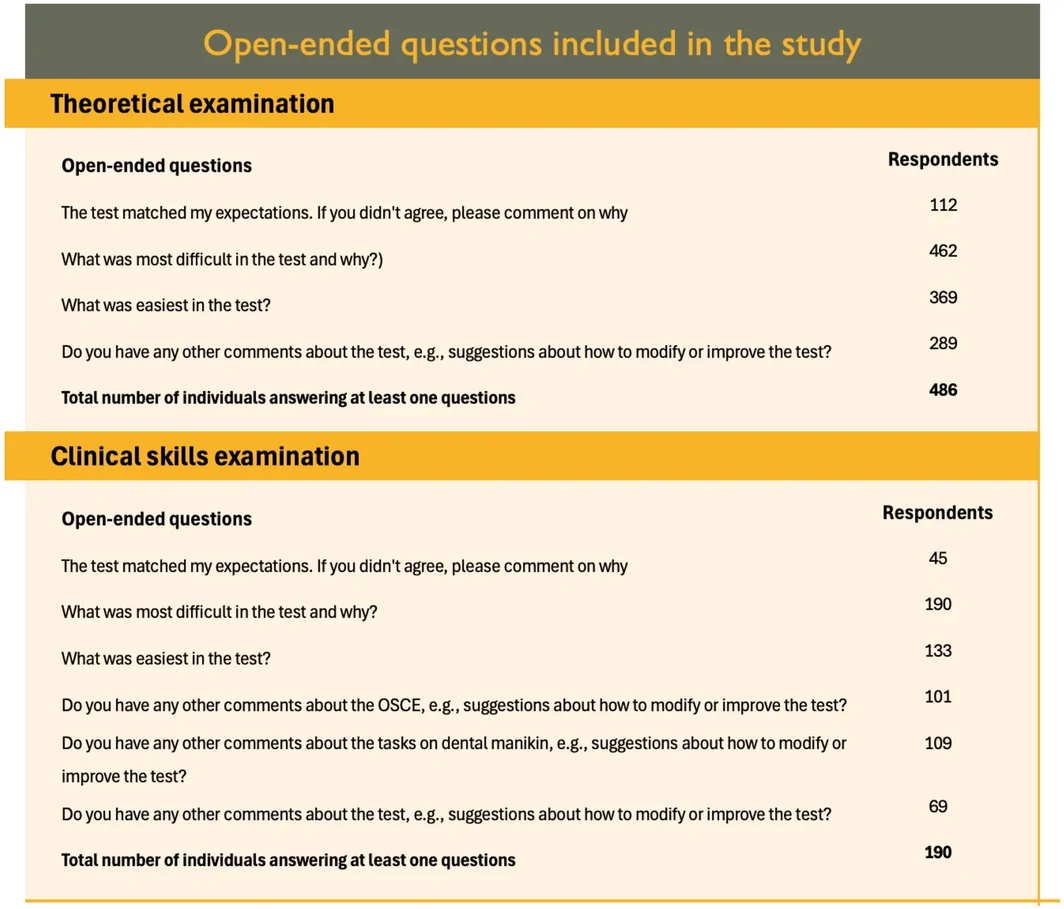
Open‐ended questions included in the study. Questions from the theoretical examination (*n* = 486) and the clinical skills examination (*n* = 190).

The survey was distributed and collected via a web‐based survey tool and was open for approximately 2 weeks. The survey was closed before the results of the examination were delivered to the participants. Five reminders were sent. The survey was not public and could only be answered with an individual login provided by email.

### 2.4 Data Analysis

Systematic text condensation was used to analyse the written responses from the participants. The method is a form of template analysis developed by Malterud, where meaningful units are sorted into predefined themes [21, 22]. The method involves four main steps: (1) obtain a total impression of the data, (2) identify and sort meaning units, (3) condense the meaning units and (4) synthesise the condensates into a coherent description of participants' experiences. The method is described in Figure 2. The analysis was data‐driven and not guided by saturation, as the purpose was to develop descriptions. This study focused on experiences expressed by the respondents, rather than underlying meanings; this was considered appropriate for analysing written answers to open‐ended surveys [20].

**FIGURE 2:**
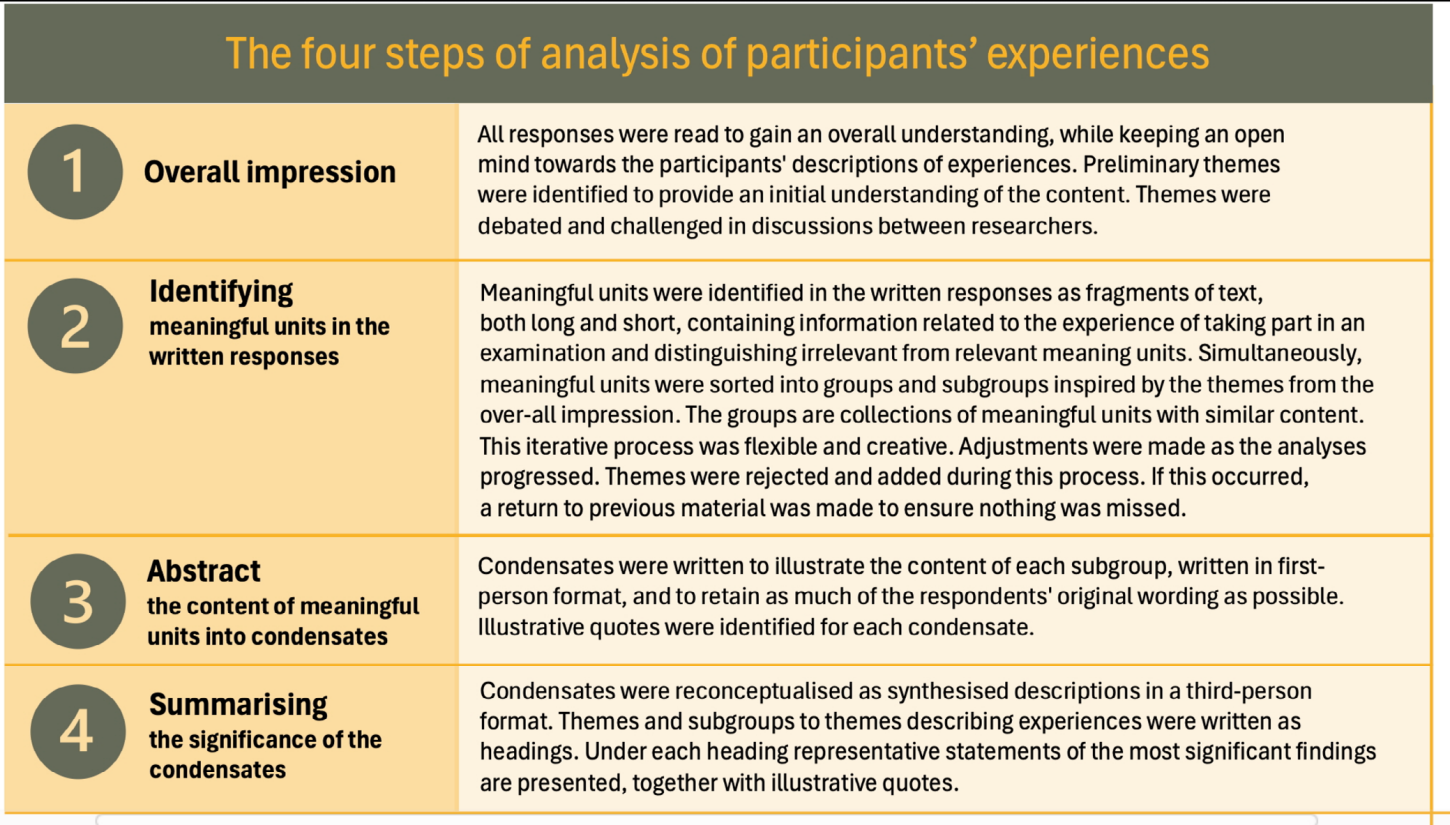
The four steps of analysis of participants' experiences, adapted from Malteruds Systematic Text Condensation: (1) gaining an overall impression, (2) identifying meaningful units, (3) abstracting the content into condensates and (4) summarising the co.

The written responses were submitted in both Swedish and English, depending on participants' language preference. All responses were analysed in their original language. Quotes presented in this article were translated into English by the research team with close attention to preserving tone and meaning. An external translator reviewed all translations to ensure linguistic accuracy and trustworthiness.

At the time of analysis, the first author had experience in planning and executing both theoretical and clinical parts of the proficiency test. The last author had direct involvement as principal examiner. The second author is a lecturer with previous experience of the method and only recently involved in the proficiency test. The third author was not involved in the recertification examinations but is experienced in dental education and qualitative research methodologies. All authors have a background in dentistry, with the first and third author being registered dental hygienists, and the second and last author being dentists. Reflexivity was actively integrated into the research process, with continuous reflection on roles, experiences and potential biases. The diversity of professional backgrounds—within and across levels of experience—contributed to a richer analysis. The first, second and fourth authors initially read all responses independently to gain an overall impression of the material. All authors participated in subsequent steps of the analysis, including identifying meaning units, developing condensates and transforming condensates into synthesised descriptions in third‐person format. Notes were taken throughout the process, and an audit trail was maintained by saving successive versions of coding documents, allowing for transparency in analytic decisions. Microsoft Word and Excel were used to manage and organise the data throughout the analysis.

### 2.5 Ethical Considerations

Ethical approval for this study was granted by the Swedish Ethical Review Authority (Protocol identification number: 2019–06028). Written consent was collected from the participants, who were informed that their responses would be included in the research. They were therefore given the choice to consent or decline the use of their responses for research purposes. Some answers included personal experiences, not directly connected to survey questions. Researchers ensured that no quotes could identify individual participants during analysis and presentation of results.

## 3 Results

Participants' experiences were summarised under **one core theme**: *challenges in demonstrating competence*. The theme was divided into four subthemes: (a) *preparing for the exams, (b) relevance of assessments, (c) stress* and *(d) language barriers*, each representing participants' experiences of *challenges* in demonstrating competence. An overview of the theme, subthemes, corresponding meaningful units and quotes from participants is presented in Figure 3.

**FIGURE 3:**
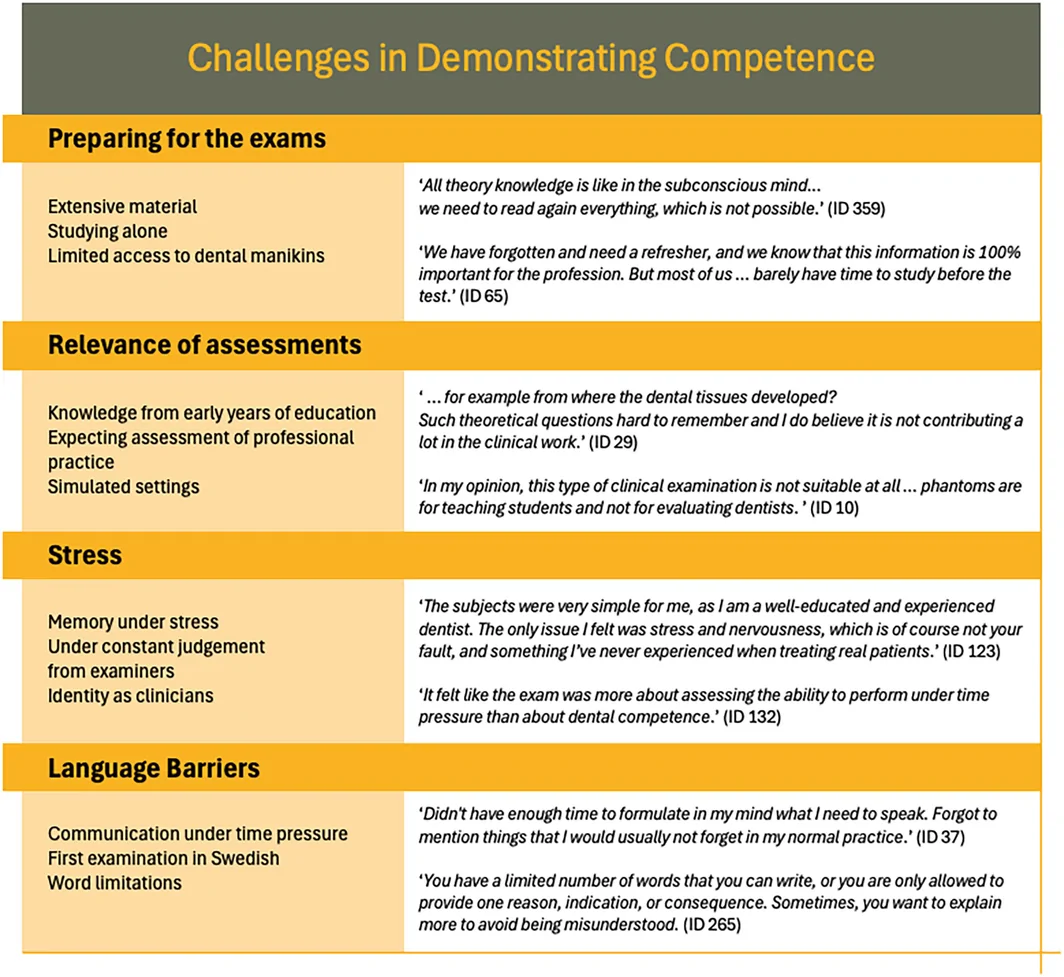
Overview of the core theme Challenges in Demonstrating Competence, with subthemes. Each subtheme is illustrated by meaningful units of participants' experience and representative quotes.

### 3.1 Preparing for the Exams

Preparing alone was described as particularly challenging. The recommended course literature was regarded as overwhelming and hard to navigate without support. The participants described a lack of structured guidance, which left them feeling unprepared and uncertain about how to approach the extensive preparatory material and the practical arrangements required for the proficiency examinations within the recognition process. In addition, an extended absence from the profession made preparation difficult, as participants described a need to refresh both basic theoretical knowledge as well as Swedish guidelines, which differed from those in their home countries. Financial pressure and limited time were further challenges for effective preparation.

The participants expressed that the difference between working on dental manikins and real patients made it challenging to prepare for the clinical skills assessments, even if they had access to a clinic for practice. Participants expressed a need for targeted recommendations from the test provider to help them focus on the most relevant material. They also highlighted a need for direction concerning Swedish guidelines, which differed from what they were familiar with in their home countries. To prepare for the practical exam, reduce stress and boost confidence, the participants suggested access to an online course with clear examples of acceptable procedures as well as access to the materials used in the exam.

### 3.2 Relevance of Assessments

Participants questioned the relevance of certain aspects of the assessments and described a misalignment between the exam content and real‐world clinical practice. In the theoretical examinations the participants were surprised by the nature of the questions, which sometimes covered areas of knowledge typically learned in the early years of dental education or specialist training, that is, theoretical knowledge not directly applicable to everyday dental practice, which limited their ability to demonstrate their competencies. This perceived misalignment created a sense of not being able to show their knowledge in dentistry, as they had expected a focus on clinical knowledge. Participants expressed that items and tasks often focused on minor details that did not reflect the most important and relevant areas in dentistry, which failed to give them the opportunity to demonstrate their actual competence in dentistry. The complexity of clinical decision‐making in some questions, where multiple treatment options might be valid, was sometimes perceived as confusing. These questions were at times interpreted as riddles rather than a test of knowledge. Participants moreover pointed out that choosing a single correct answer from multiple options does not reflect real‐world clinical practice. Participants also described confusion in the test environment, particularly in the OSCE stations, where their interpretation of the descriptions did not always match the tasks encountered. The participants expressed that tools and equipment used in the exam were of high quality, but they found it challenging to work on a dental manikin and to use unfamiliar instruments. Consequently, participants were concerned that the tests did not effectively measure their competence as practising dentists. Participants described appreciating questions based on patient cases and real clinical situations from daily dental practice, rather than theoretical details learned in the early years of education. At the same time, participants expressed a need for more straightforward knowledge questions. They also described an appreciation for the stations where the tasks were clear, the communication was straightforward, and the clinical questions were perceived as well‐formulated.

### 3.3 Stress

Stress was described as a factor influencing participants' performance. The high stakes nature of the recertification process was perceived as stressful, leaving participants feeling overwhelmed and unable to perform at their best. In the theoretical examinations, the participants expressed that the exams often did not cover the key subjects they had focused on, which led to frustration and increased stress. They described that stress negatively affected their ability to remember important information and perform at their best. The clinical skills examinations were described as particularly stressful, with participants feeling constantly judged by examiners. With the presence of numerous staff members, participants felt they could not show their true competence, a factor contributing to underachievement. Participants also noted that being unfamiliar with the equipment and the time pressure during the clinical exams further increased their stress and negatively affected their performance. The hands‐on demands and time constraints added to their stress and confusion, especially for those who had been away from practice for some time. The participants had different views on whether the time was sufficient. Some participants reported that the time allocated for the examinations was not enough to accurately demonstrate knowledge and skills. Participants expressed a need for a less stressful environment, like a regular educational day at the university. During the exams, participants appreciated the kindness and support from both patients and examiners. In the clinical skills examinations, participants expressed a need for extended time, as time constraints hindered them from performing tasks as they would in a typical clinical setting. However, other participants reported that enough time was allocated to complete all tasks and described the environment as friendly and stress‐free.

### 3.4 Language Barriers

Language barriers were described as a significant challenge, affecting participants' ability to fully demonstrate their competence during the exams. The participants struggled to understand exam questions and communicate effectively under time pressure in the clinical examination, particularly when taking the exam in Swedish for the first time. They described that the exams tested their language skills more than subject knowledge, which negatively affected their performance and ability to demonstrate competence. Negative feelings were directed towards questions in the theoretical examination, as some questions were experienced as manipulative in their wording, distracting participants from reaching the correct answer. In essay questions, word limits caused frustration, as they prevented participants from elaborating on their clinical reasoning. Participants expressed pride in their language skills when they were able to understand the different dialects spoken by the simulated patients. At the same time, they described challenges with unfamiliar dialects and rapid speech, which made it difficult to follow instructions and interact effectively with the simulated patients. They also described difficulties in finding the right words, which sometimes led them to forget important details they would normally recall in a regular clinical setting. Concerning language, participants expressed a need for clearer and more straightforward wording in the exams to demonstrate their competence more accurately. They also described needing more time to read and understand the questions. During clinical exams, the participants described patient communication as straightforward and the clinical questions as well‐formulated, making the language and content manageable.

## 4 Discussion

In this study we described the experiences of third‐country dentists undertaking the Swedish proficiency test and identified four key challenges including (a) preparing for the exams, (b) relevance of assessments, (c) language barriers and (d) stress. The participants described difficulties preparing for the examinations, including extensive literature that was hard to navigate without support, challenges in refreshing knowledge, limited access to material and equipment to prepare for clinical exams, financial pressure and a lack of time. These findings are consistent with previous research on overseas‐trained dentists, physicians and nurses who faced similar challenges adapting to local clinical practices in high‐stakes assessments [10, 16, 17], with similar experiences of loss of income, loss of time [9, 14, 23] and the need for structured support in re‐licencing [9, 11, 14, 16]. Previous studies of the theoretical examinations showed that older candidates scored significantly lower [5]. This may be an indication that younger dentists, being closer to their dental education, can more easily recall and refresh their undergraduate education, whereas more experienced dentists find this more difficult. Moreover, participants described it as difficult to prepare for the clinical skills examination; although some had opportunities for clinical practice in dental clinics, they still lacked access to dental manikins. The proficiency test is designed to evaluate whether healthcare professionals have the knowledge and skills required of a licenced practitioner. It is important to clarify that the Swedish National Board of Health and Welfare's Proficiency test is not an educational pathway to licensure. Therefore, if participants approach it with expectations of an educational pathway, the rigorous assessment focus may appear to lack sufficient preparatory material and opportunities to practice. However, all proficiency tests aim to be transparent about what is assessed and why. Consequently, participants are provided with extensive information about the test, as well as references to relevant study materials. Moreover, theoretical and practical examinations, along with their answer keys, are made publicly available after the test. For those requiring more preparation than self‐study allows, the 1‐year additional training programme at a Swedish university is recommended.

The participants expressed language as a barrier in various forms: when preparing for examinations, reading and understanding questions, communicating with patients and demonstrating competence—especially under time pressure. The findings align with studies of nurses and migrant physicians in Sweden, which showed that language difficulties were associated with lower pass rates in licencing examinations [16, 17]. The results of the present study support our previous findings, showing that taking the theoretical exam multiple times was a significant factor for passing [5] and that good communication skills reduced the risk of failing the clinical skills examination and the OSCE [6]. This may indicate that proficiency in both writing and speaking in a Swedish dental context is important to pass the examinations, and that such proficiencies improve over time. The results suggest that adding language support in preparation for the proficiency examinations could help participants and increase the effectiveness of the process.

Participants also experienced constant stress, related to perceived lack of time, unclear instructions and unfamiliar equipment used in clinical examinations. This mirrors the experiences of nurses undergoing recognition processes in Sweden, who reported stress, anxiety, uncertainty and frustration when taking the proficiency test [14]. In the present study, the participants reported that the support and kindness shown by the test provider during the examinations helped reduce stress and enabled them to demonstrate their competencies more effectively.

The participants described items and tasks as non‐representative of real clinical situations, limiting their ability to fully demonstrate their clinical competence. These descriptions of misalignment between the examination content and their ability to demonstrate competence have not been reported in previous research on recognition processes. These expectations, seen as a misalignment, were best described in the clinical exams: although participants knew what to do and had trained for it, the simulated context and unfamiliar environment hindered them from demonstrating their true competence.

In this context, it is important to note that the assessment framework of the proficiency test is designed in alignment with Miller's hierarchical model of competence [24], which begins by assessing knowledge (‘Know’ and ‘Know how’) in the theoretical examination before progressing to the demonstration of skills in a simulated environment (‘Shows how’) in the clinical skills examination. Professional practice (‘Does’), the top of Miller's pyramid, is primarily evaluated during the 6‐month clinical practice that follows the theoretical and clinical exams. Consequently, the competence, in professional practice, that participants describe unable to fully demonstrate is, by design, not intended to be assessed in the theoretical and clinical skills examinations. This issue, where experienced practitioners face challenges in demonstrating clinical skills in simulated settings, is also reflected in proficiency test outcomes, as advancing age increases the risk of failing clinical skills examinations [6], challenging the assumptions that clinical experience correlates with stronger exam performance. This result may also be partly explained by previous research showing that experienced clinicians, such as dentists taking the proficiency test, often demonstrate greater efficiency than proficiency [25], which can be penalised in standardised assessment formats [26].

These findings add to the understanding of how assessment formats function in terms of efficiency and validity when applied to experienced practitioners. When practising dentists are required to demonstrate competence primarily at the lower levels of Miller's pyramid [24], performance may be influenced by familiarity with the assessment format or language skills rather than by their actual competence in professional practice. Clarifying the purpose and scope of each assessment stage and aligning tasks with the intended level of competence may improve both efficiency and validity. It can further help academics, stakeholders and test providers to avoid drawing conclusions about professional practice from assessments aligned with the lower levels of Miller's pyramid. Additionally, when designing exam items and tasks for licencing assessments of foreign‐trained dentists, participants' professional experience should be considered, as they are practising dentists rather than students. The findings may prompt reflection among dental schools and academics on the essential competence required at the point of licensure, and on how well curricula and assessments align with these expectations. Such reflections are important for moving towards greater harmonisation of expectations and assessment principles across European dental education. Clear and shared standards for licensure would help define essential competence for a European dentist, supporting patient safety both within individual countries and across the European Union.

## 5 Limitations

The questionnaires used in the study were not validated, and the survey format did not allow for follow‐up questions, which may affect the reliability of the findings. However, the survey was created in collaboration with experienced researchers at the Department of Learning, Informatics, Management and Ethics, Karolinska Institutet, Huddinge, Sweden and the consistency of the survey questions over time provides a degree of stability. Self‐reported survey data also have limitations, as respondents may have different experiences from non‐respondents. Factors such as the willingness to share experiences and the belief that feedback would be valued likely influenced participation [27]. Since the survey sought suggestions for improvement, there may be a bias towards negative feedback, as satisfied participants might not feel compelled to respond, particularly to open‐ended questions, resulting in more critical responses and under‐representation of positive aspects. Non‐respondents may have chosen not to participate due to doubts about whether their feedback would have an impact on change, or because of language barriers if they were not fluent in Swedish or English. Reminders were sent to encourage participation and anonymity was ensured to promote honest feedback. Although the participants' experiences are presented truthfully, they do not fully represent the diverse group of foreign‐trained dentists undergoing the recognition process. Nonetheless, the insights from this study contribute to understanding the experiences of foreign‐trained healthcare professionals and provide a basis for future improvements.

Concerning the methods used in the present study, there can be multiple valid interpretations in qualitative analysis. Through reflexivity and iterating back and forth between the stages of analysis and working alongside experienced co‐authors, the interpretations and syntheses were challenged and re‐evaluated. The research team had diverse knowledge about the examinations and methods of analysis, providing multiple perspectives throughout the process. To mitigate the risk of losing the individual context of data, the team recontextualised by continually referring to the original text and validating interpretations at each step [20, 28]. To ensure the credibility of the quotes, an external translator was engaged to translate quotes from Swedish to English. To validate the findings, future research should involve participants, allow for follow‐up and include participants in results and conclusions to provide a deeper understanding of the perceived challenges and needs. Longitudinal studies are suggested to track dentists' progress and well‐being after recertification to evaluate long‐term impacts. Additionally, research should explore how targeted language support influences experiences of the recognition process. Comparative studies across countries in a European context are suggested to identify best practices and guide improvements. Consequently, this study's findings are relevant, not only for Sweden but also for other nations seeking to optimise their recognition processes in line with European standards.

## 6 Conclusion

Participants confirmed known challenges in the recognition process while also highlighting a novel concern: they described the theoretical and clinical examinations, modelled on undergraduate assessments, as designed for students and as non‐representative of real clinical situations, which they described as hindering their ability to demonstrate competence in professional practice.

## Funding

The authors have nothing to report.

## Conflicts of Interest

The authors declare no conflicts of interest.

## Supporting information


**Data S1:** eje70124‐sup‐0001‐Supinfo.docx.

## Data Availability

The data that support the findings of this study are available from the corresponding author upon reasonable request.
