# Supplementary material for: Foreign‐Trained Dentists' Experiences of the Swedish Proficiency Test for Recognition of Dental Qualifications
Source: Eur J Dent Educ. 2026 Feb 27;30(4):1835–43. doi: 10.1111/eje.70124 (PMC13686671; doi:10.1111/eje.70124)
Supplement: Supplementary file 1 [file EJE-30-1835-s001.docx]

**Appendices**

## A. **Survey after the theoretical examination**

Nyligen deltog du i kunskapsprovet för tandläkare med utbildning från länder utanför EU/EES. Vi är nyfikna på dina upplevelser av provet och skulle gärna ta del av dina synpunkter. Dina kommentarer skulle hjälpa oss att förbättra provet och vi skulle därför vara tacksamma för din input. Dina svar kommer att behandlas konfidentiellt och påverkar inte på något sätt dina resultat på provet.


Recently you participated in a proficiency test for dentists educated outside the EU/EEA/Switzerland. We are curious about your experiences of the test and would welcome your opinions on it. Your comments could help us improve the test and we would therefore be grateful for your input. Your answers will be treated as confidential, and they will not in any way affect your test results.

1. Provet överensstämde med mina förväntningar
   The test matched my expectations
2. Om du inte höll med, kommentera gärna varför:
   If you disagreed, please comment why:
3. Jag är nöjd med min prestation på provet
   I am satisfied with my performance on the test
4. Det fanns tillräckligt med tid till att besvara provfrågorna
   There was enough time to answer all the test questions
5. Frågorna i provet var överlag lätta
   Overall, the tasks in the test were easy
6. Språket i provet var lätt att förstå
   The language used in the test was easy to understand
7. Provet handlade om ett antal olika ämnen, presenterade flera patientfall och inkluderade flera olika typer av uppgifter. Vad var svårast på provet och varför?
   The test covered a number of different subjects, presented several patient cases and included different kinds of tasks. What was most difficult in the test and why?
8. Vad var lättast på provet?
   What was easiest in the test?
9. Hur relevant var del 1, för att bedöma tandläkarkompetens?
   How relevant was part 1, to assess competence of a dentist?
10. Hur svår var del 1?
    How difficult was part 1?
11. Hur bedömer du din egen prestation i del 1?
    How would you assess your own performance in Part 1?
12. Hur relevanta var uppgifterna om den vetenskapliga artikeln för att bedöma tandläkarkompetens?
    How relevant was the part Vetenskaplig artikel: , to assess competence of a dentist?
13. Hur svåra var frågorna rörande den vetenskapliga artikeln?
    How difficult was the questions concerning Vetenskaplig artikel?
14. Hur bedömer du din egen prestation på uppgifterna rörande den vetenskapliga artikeln?
    How do you rate your own performance on the tasks relating to the scientific article?
15. Hur relevant var del 2 för att bedöma tandläkarkompetens?
    How relevant was part 2, to assess competence of a dentist?
16. Hur svår var del 2?
    How difficult was part 2?
17. Hur bedömer du din egen prestation i avsnittet Del 2?
    How do you rate your own performance in Part 2?
18. Har du några andra kommentarer om provet, tex förslag på hur du tycker att provet borde modifieras eller förbättras?
    Do you have any other comments about the test, e.g., suggestions about how to modify or improve the test?
19. I vilket land studerade du till tandläkare?
    In which country did you study to become a dentist?
20. Hur många år har du arbetat som tandläkare?
    How many years of work experience do you have as a dentist?
21. Hur många gånger har du gjort det här provet?
    How many times have you taken this exam?
22. Jag tillåter att svaren på frågorna används till forskning. Dina svar kommer att behandlas konfidentiellt. Dina svar påverkar inte på något vis ditt resultat på provet. Vid presentation av eventuella resultat kommer alla data att vara anonymiserade.
    I accept that the answers may be used for research. Your answers will be treated confidentially. Your answers will not in any way affect your test results. In results, all data will be anonymized.

Vi tackar dig för dina svar!
We thank you for your input!

## B. **Survey after the practical examination**

Nyligen deltog du i det praktiska kunskapsprovet för tandläkare med utbildning från länder utanför EU/EES. Vi är nyfikna på dina pplevelser av provet och skulle gärna ta del av dina synpunkter. Dina kommentarer skulle hjälpa oss att förbättra provet och vi skulle därför vara tacksamma för din input. Dina svar kommer att behandlas konfidentiellt och påverkar inte på något sätt dina resultat på provet.

Recently you participated in a proficiency test for dentists educated outside the EU/EEA/Switzerland. We are curious about your experiences of the test and would welcome your opinions on it. Your comments could help us improve the test and we would therefore be grateful for your input. Your answers will be treated as confidential, and they will not in any way affect your test results.

1. Provet överensstämde med mina förväntningar
   The test matched my expectations
2. Om du inte höll med, kommentera gärna varför
3. Jag är nöjd med min prestation på provet
   I am satisfied with my performance on the test
4. Det fanns tillräckligt med tid till att genomföra provet
   There was enough time to finish the test
5. Provet var överlag lätt
   Overall, the tasks in the test were easy
6. Språket i provet var lätt att förstå
   The language used in the test was easy to understand
7. Provet handlade om ett antal olika ämnen, presenterade flera patientfall och inkluderade flera olika typer av uppgifter. Vad var svårast på provet och varför?
8. The test covered a number of different subjects, presented several patient cases and included different kinds of tasks. What was most difficult in the test and why?
9. Vad var lättast på provet?
   What was easiest in the test?
10. Hur relevant var OSCE för att bedöma tandläkarkompetens?
    How relevant was OSCE to assess competence of a dentist?
11. Hur svårt var OSCE?
    How difficult was OSCE?
12. Hur bedömer du din egen prestation på OSCE
    How do you rate your own performance in OSCE
13. Har du några kommentarer om OSCE, tex förslag på hur du tycker att provet borde modifieras eller förbättras?
    Do you have any other comments about the OSCE, e.g., suggestions about how to modify or improve the test?
14. Hur relevant var PreKlin för att bedöma tandläkarkompetens?
    How relevant was PreKlin to assess competence of a dentist?
15. Hur svårt var PreKLin?
    How difficult was PreKlin?
16. Hur bedömer du din egen prestation på PreKlin
    How do you rate your own performance in PreKlin
17. Har du några kommentarer om PreKlin, tex förslag på hur du tycker att provet borde modifieras eller förbättras?
    Do you have any other comments about the PreKlin, e.g., suggestions about how to modify or improve the test?
18. Har du några andra kommentarer om provet, tex förslag på hur du tycker att provet borde modifieras eller förbättras?
    Do you have any other comments about the test, e.g., suggestions about how to modify or improve the test?
19. I vilket land studerade du till tandläkare?
    In which country did you study to become a dentist?
20. Hur många år har du arbetat som tandläkare?
    How many years of work experience do you have as a dentist?
21. Hur många gånger har du gjort det praktiska provet?
    How many times have you participated in the practical exam?
22. Jag tillåter att svaren på frågorna används till forskning. Dina svar kommer att behandlas konfidentiellt. Dina svar påverkar inte på något vis ditt resultat på provet. Vid presentation av eventuella resultat kommer alla data att vara anonymiserade.
    I accept that the answers may be used for research. Your answers will be treated confidentially. Your answers will not in any way affect your test results. In results, all data will be anonymized.

Vi tackar dig för dina svar!
We thank you for your input!
